# The Quantitative Detection of Cystatin-C in Patient Samples Using a Colorimetric Lateral Flow Immunoassay

**DOI:** 10.3390/bios14010030

**Published:** 2024-01-08

**Authors:** Santosh Kumar Bikkarolla, Kavipriya Venkatesan, Yeddula Rebecca Revathy, Sowmya Parameswaran, Subramanian Krishnakumar, Dhananjaya Dendukuri

**Affiliations:** 1 Achira Labs, 66b, 13th Cross Rd, Dollar Layout, 3rd Phase, J. P. Nagar, Bengaluru 560078, India; 2 Vision Research Foundation, Chennai 600006, India

**Keywords:** gold nanoparticles, lateral flow immunoassay, Cystatin-C, patient-whole blood, serum-samples

## Abstract

A colloidal gold-based lateral flow immunoassay was developed for the rapid quantitative detection of Cystatin-C in serum and whole blood. This device has an assay time of 15 min, making it a convenient point-of-care diagnostic tool. The device has a quantification range spanning from 0.5 to 7.5 µg/mL, with a lower limit of detection at 0.18 µg/mL. To validate its accuracy, the test was compared to a standard nephelometric immunoassay, and the results exhibited a robust linear correlation with an adjusted r^2^ value of 0.95. Furthermore, the device demonstrates satisfactory levels of analytical performance in terms of precision, sensitivity, and interference, indicating its potential for precise Cystatin-C quantification, particularly in renal-failure patients. Notably, the Cystatin-C-LFA device also demonstrates satisfactory stability, as a 30-day accelerated stability study at 50 °C showed no change in the device performance, indicating a long shelf life for the product when stored at room temperature.

## 1. Introduction

Acute kidney injury (AKI) or chronic kidney disease (CKD) are characterized by a reduction in glomerular filtration rate (GFR) [1]. In the course of CKD, monitoring GFR is crucial, as it is used to estimate functional nephron loss and to guide the diagnosis and treatment of kidney diseases [2]. At present, serum creatinine levels are used for the evaluation of GFR [3,4]. However, serum creatinine is not an ideal marker for the estimation of GFR, as the levels of creatinine increase only after a 30 to 50% reduction in GFR [5]. This means that any mild or moderate reduction in GFR cannot be detected using creatinine levels, leading to late detection of kidney failure. In addition, creatinine levels can also vary due to the patient’s gender, age, muscle mass, and diet, leading to false-positive or false-negative results [6,7,8].

Cystatin-C is a non-glycated protein with a molecular weight of 13.3 kDa, belonging to cystatin protease inhibitors [9]. It is produced by all nucleated cells in the body [10]. Cystatin-C in systemic circulation can only be cleared by glomerular filtration and can be reabsorbed through the proximal convoluted tubule, followed by catabolism, without returning to the bloodstream. This indicates that only glomerular filtration determines the concentration of Cystatin-C in the blood and does not rely on any other external factors, such as muscle mass, age, diet, and gender. It also suggests that Cystatin-C is a better marker reflecting the changes in the GFR [11,12]. In a healthy human, the concentration of Cystatin-C in whole blood is in the range of 0.51–1.5 µg/mL, which can increase up to ten times the normal levels as GFR decreases when kidney failure occurs. Symptoms such as overhydration, hyperkalemia, azotemia, and metabolic acidosis will occur [13,14]. 

Various immunoassay techniques are employed for the quantification of Cystatin-C, including particle-enhanced nephelometric immunoassay (PENIA), turbidimetric immunoassay (PETIA), enzyme-linked immunosorbent assay (ELISA), and radioimmunoassay (RIA) [13,15,16,17,18]. Of these, PENIA and PETIA are commonly utilized for serum Cystatin-C quantification. Nevertheless, these methods involve intricate and precise laboratory procedures, including multiple incubations, washings, and sample pretreatment steps, which limit their practical use in society and markets [19]. In contrast, lateral flow immunoassays (LFAs) are regarded as point-of-care sensors due to their simplicity, user-friendliness, and cost-effectiveness. These assays employ colloidal gold nanoparticles (AuNPs) as labels, producing distinct colored lines that can be assessed with a portable reader to determine the analyte concentration [20,21,22,23]. Unlike traditional fluorescent LFAs, the use of AuNPs as reporters overcomes shortcomings such as poor stability, photobleaching, and the need for expensive detection systems [24]. While there are reports on the quantitative detection of Cystatin-C in serum and urine using fluorescence LFAs [25,26], there have been limited investigations into the colorimetric detection of Cystatin-C, and a comprehensive validation of these devices is still lacking [27].

**Figure 1:**
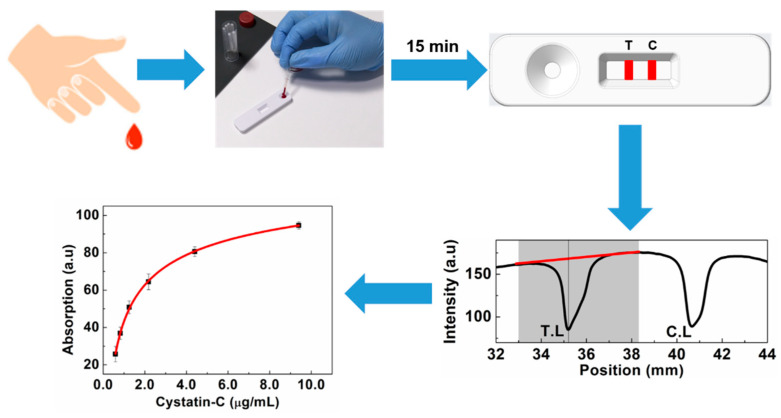
The schematic of the experimental procedure in analyzing the developed LFA strip. The shaded area represents the tolerance and the red line is for the baseline correction.

In this study, we developed a colloidal gold-based lateral flow immunoassay device for the quantitative detection of Cystatin-C with an assay time of 15 min with the experimental procedure shown in Figure 1. The developed device exhibited a range of quantification from 0.5 µg/mL to 7.5 µg/mL and a lower limit of detection of 0.18 µg/mL. These characteristics make the developed device suitable for point-of-care testing and can also help to spread the wide usage of Cystatin-C as a renal failure marker. The performance of the Au-LFA device was investigated by performing accuracy, stability, analytical sensitivity, specificity, and repeatability studies. 

## 2. Materials and Methods

### 2.1. Materials

Nitrocellulose (NC) membrane roll (150CNPH-N), backing cards (LP-25), and cassettes (device 3) were purchased from MDI, Ambala Cantt, India. Absorbent pad (Grade 243), glass fiber (8951), and blood separation pad (HV Plus, 1668) were purchased from Ahlstrom-Munksjö, Bethune, SC, USA. The monoclonal mouse anti-Cystatin-C antibody (Cyst 24cc, Hytest, Turku, Finland) and monoclonal mouse anti-Cystatin-C antibody (Cyst 28, Hytest) were used as capture and detection antibodies, respectively. Recombinant Cystatin-C antigen (8CY5) was purchased from Hytest, Turku, Finland. The polyclonal goat anti-mouse IgG antibodies (41-GM25) were used as control-line antibodies and obtained from Fitzgerald Industries International, Acton, MA, USA. Both Cystatin-C whole blood and serum samples were obtained from Padmashree Diagnostics, Bangalore, India. A human plasma bag of volume 200 mL has been obtained from Jeeva Voluntary Blood Bank & Diagnostics, Bangalore, India. Carboxyl-coated gold nanoparticles conjugation kit (ab269942) was purchased from Abcam, Cambridge, UK. 1-Ethyl-3-(3-dimethyl aminopropyl) carbodiimide (EDC, 03449-1 G), phosphate-buffered saline (PBS), 10X tris buffer solution (TBS), bovine serum albumin (BSA), Tween-20, and carbon black (242276) were purchased from Sigma Aldrich, Gillingham, UK.

### 2.2. Instruments

A ZX1010 dispense platform from Biodot (Irvine, CA, USA) was used to deposit the antibodies onto the NC membrane and spray the conjugate onto the conjugate pad. A high-speed guillotine cutter from Prahas Healthcare, Vadodara, India was used to cut the assembled materials. An ESEQuant Flex reader from Dialunox GmbH, Stockach, Germany was used to analyze the test lines. An infinite M Plex plate reader from Tecan (Männedorf, Switzerland) was used to obtain the UV-Vis spectra. 

### 2.3. Antibody Conjugation onto Gold Nanoparticles

The Cystatin-C detection antibody was purified using an Amicon filter unit, with pore size 10 kDa, to remove any amine-terminated molecules that might interfere with the conjugation process, and the purified antibodies were resuspended in a 10 mM potassium phosphate buffer of 7.4 pH. A conjugate of AuNP-Cystatin-C antibody was prepared in accordance with the recommended protocol provided by Abcam, specifically for the product with the code ab269942 [28]. In brief, AuNPs functionalized with the carboxyl group were used to covalently bind Cystatin-C antibodies by using water-soluble EDC. Then, 20 µL of 0.1 mg/mL Cystatin-C detection antibodies were mixed into 50 µL of AuNP suspension (40 OD) followed by the addition of 20 µL of 1 mM EDC. The resultant solution was incubated for 30 min at room temperature. Then, 1 mL of 1XTBS (containing 0.05% Tween) was added to the mixture and centrifuged at 8000 rpm at 4 °C for 10 min. The supernatant was carefully removed, and the pellet of the AuNP-antibody was resuspended in 90 µL of 1XTBS, 0.5% BSA, 2% Sucrose, and 0.05% Tween to obtain 20 OD or 5 OD of the conjugate. 

### 2.4. Preparation of Depleted Serum, Calibrators, Clinical Serum, and Blood Samples

Stripping of human plasma was performed by using carbon black to prepare the Cystatin-C-depleted serum [29]. Human plasma pH was adjusted to 9.0 by using 1 M NaOH and collected in 50 mL centrifuge tubes. To 30 mL of plasma, 6 g of carbon black was added and incubated for 16 h at 4 °C. After incubation the charcoal-treated plasma was centrifuged at 8000 rpm for 30 min at 4 °C to remove carbon black. The supernatant was collected in a fresh tube and centrifuged again at 8000 rpm for 30 min at 4 °C, and the collected supernatant was filtered through a 0.45 µm and 0.2 µm syringe filter to remove any carbon particles. The filtrate was collected and stored at 4 °C for further use. Cystatin-C antigen was spiked in 500 µL of depleted serum to prepare calibrators at 10 µg/mL, 5 µg/mL, 2.5 µg/mL, 1.5 µg/mL, 1.0 µg/mL and 0.5 µg/mL concentrations. Cystatin-C levels in the prepared calibrators were evaluated using the Atellica NEPH-630 analyzer to determine the Cystatin-C levels accurately. After calibrators evaluated through Atellica NEPH-630, Cystatin-C levels were measured at 9.4 µg/mL, 4.4 µg/mL, 2.18 µg/mL, 1.25 µg/mL, 0.82 µg/mL, and 0.59 µg/mL in the calibrators. A total of 93 serum and 21 whole blood samples were obtained from Padmashree Diagnostics. The concentration of these patient samples was measured using the nephelometry technique, and the values are presented in Table S1. Serum samples were collected using vacutainers containing clot activator and stored at −20 °C to prevent Cystatin-C degradation. All serum samples were stored at −20 °C to prevent degradation of Cystatin-C. It is worth mentioning that all the serum samples did not show any degradation in Cystatin-C concentration for more than three months. Blood samples were collected using K2-EDTA-coated vacutainers and were stored at 4 °C for a maximum of one day prior to measurement. The study was approved by the ethical committee of Padmashree Diagnostics at Bangalore.

### 2.5. Fabrication of LFAs

Cystatin-C capture antibodies and control line antibodies were dispersed in a printing buffer (10 mM PBS, 1% sucrose, pH: 7.4) at a concentration of 2 mg/mL and 1 mg/mL. Both the antibodies were dispensed onto the NC membrane using a Biodot (ZX1010) dispense platform at a flow rate of 1 µL/cm to obtain a test line and control line width of 1 mm. The NC membrane was dried in an oven at 37 °C for 120 min. Gold conjugate (5 OD) was sprayed onto a glass fiber pad at a flow rate of 10 µL/cm and dried in an oven at 37 °C for 120 min. Finally, the NC membrane, conjugate pad, absorbent pad, and sample pad were manually assembled on a plastic backing card and cut into 4.75 mm test strips with a guillotine cutter. The LFA strips were enclosed in a lateral flow assay cassette for easy handling and sample loading.

### 2.6. Colorimetric Lateral Flow Immunoassay Procedure

A total of 5 µL of Cystatin-C serum samples or 8.5 µL of blood samples were added into 495 µL of dilution buffer (10 mM PBS, 1% BSA, and 1% Tween-20) and mixed thoroughly. Subsequently, 60 µL of the mixture was loaded onto the sample port of the cassette and wicked towards the absorbent pad with capillary forces. After the assay time, the device was inserted into the LFA reader to measure the peak area of the test and control lines under the illumination of 530 nm wavelength. Only the test line peak area was considered when obtaining calibration curves and for measurements thereafter.

### 2.7. Validation of Developed LFA

The performance of the developed device is characterized by recovery, stability, reproducibility, and interference. The recovery and interference tests were applied to investigate the matrix effects and individual components of the blood. Accelerated stability tests were performed to determine the shelf life of the developed devices. The depleted serum was tested 10 times to obtain the mean and standard deviation. The concentration corresponding to mean +3 × SD is considered as the limit of detection (LOD) and the concentration corresponding to mean +10 × SD is considered as the limit of quantification (LOQ) [25,30]. 

### 2.8. Measurements with Atellica NEPH-630

The FDA-approved Atellica NEPH-630 analyzer is widely used as the gold standard for measuring Cystatin-C concentrations in serum samples. It is based on the principle that a dilute suspension of small particles will scatter light passed through it rather than simply absorbing it. A sample containing the target antigen is mixed with a specific antibody labeled with a particle. The antibody binds selectively to the antigen, forming an antigen–antibody complex. The presence of antigen–antibody complexes causes the light to scatter. The extent and pattern of scattering depend on the concentration of the antigen–antibody complexes. The intensity of scattered light from the sample is then compared to the calibration curve to determine the concentration of the antigen in the original sample. Atellica NEPH-630, which is an FDA approved analyzer, is considered as a gold standard technique for obtaining Cystatin-C concentrations in serum samples. 

## 3. Results and Discussion

**Figure 2:**
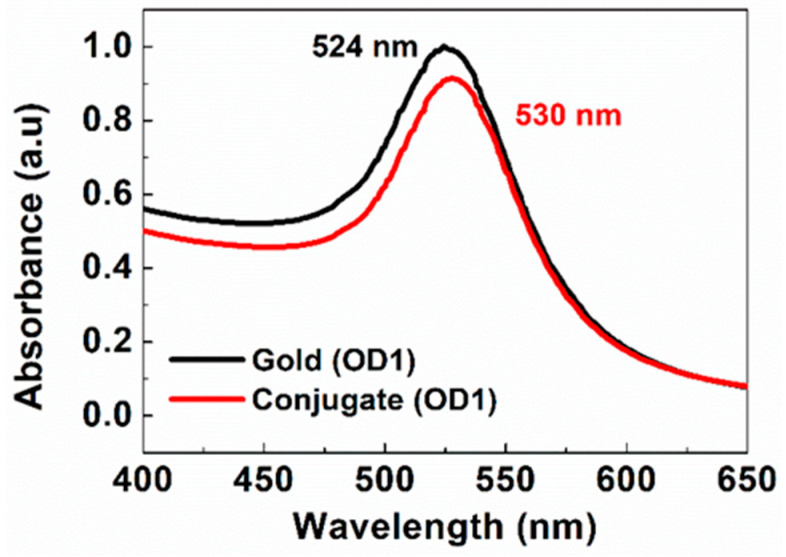
The UV-VIS spectra of 40 nm gold nanoparticles before and after conjugation.

UV-VIS absorption spectra were measured to reveal the plasmonic properties and stability of the Au and Cystatin-C-Au nanoparticles. UV-VIS spectra are collected before and after the conjugation of antibodies to the gold nanoparticles. Figure 2 shows the successful conjugation of antibodies onto the gold nanoparticles where the resonance peak is shifted to the right by about 4 nm (red shifted) due to the effects of the conjugated protein on the plasmon resonance peak of the gold nanoparticle [28,31,32]. In addition, the FWHM and OD of the conjugate are the same as those of the gold nanoparticles, indicating that the conjugate consists of monodispersed gold nanoparticles attached to the detector antibody.

**Figure 3:**
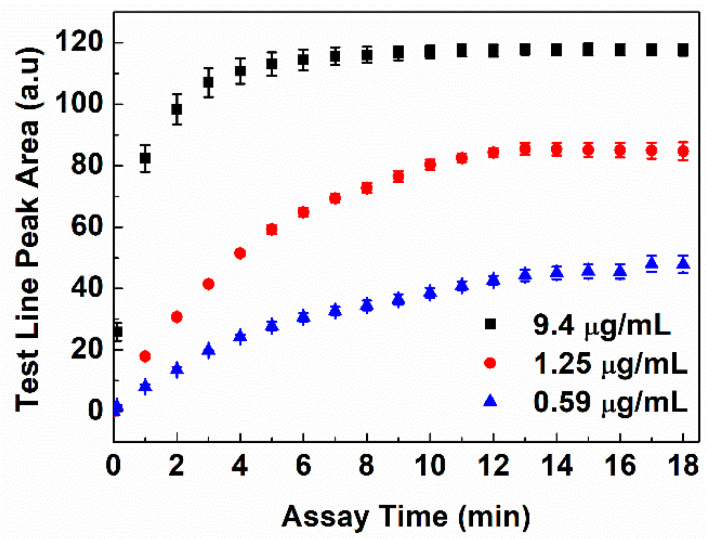
The optimization of assay time with three calibrators at 9.4 µg/mL, 1.25 µg/mL, and 0.59 µg/mL.

**Figure 4:**
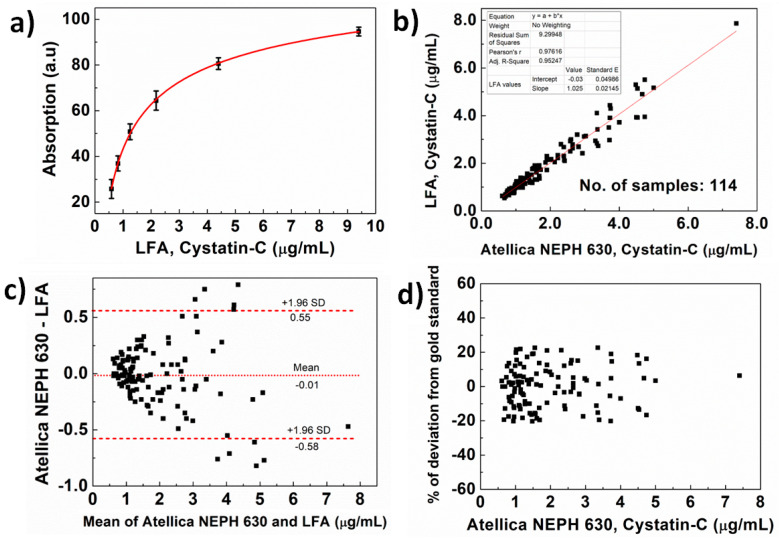
(**a**) The calibration curve of the developed LFA obtained with serum calibrators for each point was obtained with three duplications. (**b**) The linear correlation between the developed LFA and Atellica NEPH-630. (**c**,**d**) The Bland–Altman plot obtained with developed LFA and Atellica NEPH-630.

The developed LFA is operated as a colorimetric sandwich immunoassay with gold nanoparticles as the reporter nanoparticles. Figure 1 shows the schematic of the experimental procedure used to analyze the test lines of the developed device. Briefly, the sample buffer is added to the blood separation pad and migrates to the conjugate pad via capillary forces. The Cystatin-C in the sample buffer specifically binds to the Au-anti-Cys-C ab and forms antigen–antibody complexes, which move forward on the NC membrane and are captured at the test and control lines. The LFA device is inserted into the colorimetric reader to obtain the peak area of the test and control lines. As the concentration of the Cystatin-C increases the test line area increases, whereas the control line area decreases as the concentration of Cystatin-C increases. Assay time is one of the most important parameters that affect the quantification of the LFA testing. Assay time has been optimized by studying the change in the peak area over time at Cystatin-C concentrations of 9.4 µg/mL, 1.25 µg/mL, and 0.59 µg/mL, as shown in Figure 3. It has been observed that test line intensity at Cystatin-C concentration of 9.4 µg/mL was saturated after 8 min and remained constant up to 18 min. In the case of a Cystatin-C concentration of 1.25 µg/mL, test line intensity was saturated after 12 min and remained constant for up to 18 min. For a Cystatin-C concentration of 0.59 µg/mL, test line intensity was saturated after 14 min and remained constant up to 18 min. This indicates that, with an assay time of 15 min, test line intensities at all clinically relevant concentrations are completely developed, and remain saturated, therefore 15 min is chosen as the assay time. Calibration curves were obtained with the developed device by using calibrators across the concentration range from 0.59 µg/mL to 9.4 µg/mL in triplicate, using an aforementioned procedure. The calibration curve is obtained by plotting the test line area versus the concentration of Cystatin-C (Figure 4a), fitted by a sigmoidal curve, and represented by Equation (1).

(1) $$y=141.58+\frac{\left(-647.50\right)}{\left(1+{\frac{x}{\left(0.095\right)}}^{0.37}\right)}$$

The LOD and LOQ derived from the aforementioned procedure are obtained as 0.18 µg/mL and 0.39 µg/mL, indicating the developed test is suitable for determining the Cystatin-C in a clinically relevant range. To evaluate the clinical performance of the developed Au-based LFA device, 114 samples that include both serum and blood were measured with the developed device. Samples were obtained from renal-failure and diabetic retinopathy patients with concentrations ranging from 0.61 µg/mL to 7.4 µg/mL; however, most of the samples were in the range of 0.61 to 3.0 µg/mL indicating the need for the development of methods for accurate determination of Cystatin-C levels within this range. All the serum samples were analyzed with an Atellica NEPH-630 and the developed LFA device. In the case of blood samples, the corresponding serum samples were analyzed with the nephelometry method, and blood samples were analyzed with the LFA device. The concentrations of all the samples obtained with the Atellica NEPH-630 and LFA methods are presented in Tables S1 and S2. 

A correlation plot of the Cystatin-C concentrations determined from the LFA results is shown against the corresponding FDA-cleared method results from the Atellica NEPH-630 in Figure 4b. The results showed a linear correlation between the Atellica NEPH-630 and the developed LFA method with the equation of regression Y = 1.02 × X − 0.03 and an r^2^ of 0.95 indicating a good linear relationship between the concentrations determined by the two methods. The systematic bias was investigated with a Bland–Altman plot for the developed LFA and the Atellica NEPH-630. In the Bland–Altman plot, the difference between the two methods is plotted against the average of the two methods (Figure 4c). The plot consists of a horizontal line at the mean of difference and the limit of agreements. The limit of agreements is defined as the mean of differences plus/minus 1.96 times the standard deviation of the differences. From Figure 4c, it is clear that the samples are equally distributed on both sides of the mean, indicating that there is no positive or negative bias and the distribution is within the 95% confidence interval. In addition, it can be observed that the difference between the two methods increases as the concentration of the Cystatin-C increases. However, Figure 4d shows the Bland–Altman plot that is plotted with the percentage of deviation from the gold standard value against the gold standard value, which indicates that the Cystatin-C level estimated with the LFA device deviates by a maximum of 20% when compared with the values measured with Atellica NEPH-630 across the range of 0.5 to 7.5 µg/mL. 

The precision of the intra and inter assay was measured to evaluate the reproducibility of the developed device. As shown in Table 1, the CVs of the intra and inter-assay were 6.78 to 7.55 and 9.28 to 10.87, respectively, which are below 15%, indicating an acceptable precision for quantification of Cystatin-C. The specificity was assessed with common blood components such as hemoglobin, triglycerides, bilirubin, and rheumatoid factor. These analytes were spiked in patient serum samples at high concentrations and Cystatin-C levels were measured by using the developed device. Table 2 shows the recoveries measured by the device in the presence of the interfering agents, which are in the range of 80–12%, indicating that the developed device does not cross-react with any of the common blood components. 

**Figure 5:**
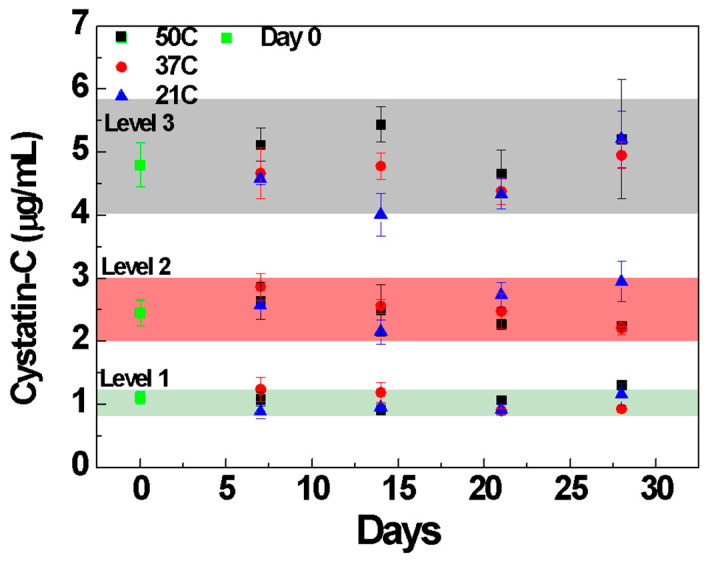
Accelerated stability summary plot; color region indicates data within 80 to 120% across levels.

To determine the shelf life of the developed device, accelerated stability studies were performed by heating the devices in sealed pouches with desiccants at 50 °C, 37 °C, and 21 °C for four weeks. Recoveries were measured at the end of every week by using three patient samples whose Cystatin-C concentrations were at 1.1 µg/mL (Level 1), 2.4 µg/mL (Level 2), and 4.8 µg/mL (level 3). Figure 5 shows the recoveries measured with the three samples in the range of 80 to 120%, indicating that the developed device has at least one year of shelf life at room temperature. 

## 4. Conclusions

In this study, we developed a colloidal gold-based lateral flow immunoassay device, which could provide a quantitative detection of Cystatin-C in human serum and whole blood within 15 min. The developed device has shown a limit of detection of 0.18 µg/mL and a range of quantification from 0.39 µg/mL to 7.5 µg/mL covering the concentration of healthy humans (0.5 to 1.5 µg/mL) and renal-failure patients. In addition, the developed device does not need either complex conjugation protocols or expensive readers which allows for the rapid development of the device and easy commercialization. In addition, the device has the potential to be used with a mobile phone reader or a scorecard to provide home-based diagnostics to track renal conditions in a point-of-care manner. 

## Figures and Tables

**Table 1:** Reproducibility of the developed LFA.

Cystatin-C Levels (µg/mL)	Average (µg/mL)	Within Run CV (%)	Between Run CV (%)	Total CV (%)
Level 1 (1.0)	0.97	6.78	9.28	10.3
Level 2 (2.43)	2.39	7.43	10.87	11.64
Level 3 (5.0)	5.17	7.55	9.76	10.58

**Table 2:** Cross-reactivity of the developed LFA.

Interferent	Concentration of Interferent	Cystatin-C (µg/mL)	Lateral Flow Result (µg/mL)	Recovery (%)
Control	-	2.0	2.2 ± 0.19	110
Hemoglobin	5 mg/mL	2.0	2.4 ± 0.089	120
Bilirubin	0.2 mg/mL	2.0	2.3 ± 0.18	115
Triglycerides	10 mg/mL	2.0	2.3 ± 0.15	115
Rheumatoid Factor	600 IU/mL	2.0	1.8 ± 0.25	80

## Data Availability

The data that support the findings of this study are available from the corresponding author upon reasonable request.

## Supplementary Materials

The following supporting information can be downloaded at: https://www.mdpi.com/article/10.3390/bios14010030/s1, Table S1 shows the serum Cystatin-C concentrations determined with Atellica NEPH-630 and the developed LFA device. Table S2 shows the blood Cystatin-C concentrations determined by the developed LFA device and the corresponding serum samples Cystatin-C concentrations determined with Atellica NEPH-630.
