# Supplementary material for: The Quantitative Detection of Cystatin-C in Patient Samples Using a Colorimetric Lateral Flow Immunoassay
Source: Biosensors (Basel). 2024 Jan 8;14(1):30. doi: 10.3390/bios14010030 (PMC10813198; doi:10.3390/bios14010030)
Supplement: Supplementary file 1 [file biosensors-14-00030-s001.zip › biosensors-2722765-supplementary.pdf]

Supporting Information

Table S1: Serum samples recovery with developed LFA.

Patient Number	Cystatin-C, Atellica-NEPH-630 (µg/mL)	Cystatin-C, LFA (µg/mL)	Recovery (%)
P1	0.61	0.63	103.28
P2	1.01	1.07	105.94
P3	1.02	1.24	121.57
P4	0.92	0.79	85.87
P5	1.13	1.2	106.19
P6	1.55	1.31	84.52
P7	0.88	0.96	109.09
P8	0.94	0.75	79.79
P9	0.93	0.95	102.15
P10	0.96	0.84	87.5
P11	1.9	1.72	90.53
P12	0.81	0.92	113.58
P13	0.76	0.85	111.84
P14	1.91	2.14	112.04
P15	1.13	1.28	113.27
P16	1.01	1.21	119.8
P17	0.99	1.11	112.12
P18	0.97	1.04	107.22
P19	0.7	0.7	100
P20	0.63	0.63	100
P21	0.97	0.94	96.91
P22	0.88	0.8	90.91
P23	0.98	0.97	98.98
P24	0.74	0.78	105.41
P25	2.4	2.13	88.75
P26	1.09	1.1	100.92
P27	1.98	2.15	108.59
P28	1.12	0.99	88.39

P29	3.39	2.73	80.53
P30	2.13	2.05	96.24
P31	2.65	2.79	105.28
P32	1.1	0.98	89.09
P33	3.03	3.14	103.63
P34	2.42	2.1	86.78
P35	0.83	0.76	91.57
P36	1.4	1.26	90
P37	0.95	0.84	88.42
P38	4.5	3.93	87.33
P39	0.8	0.66	82.5
P40	1.41	1.19	84.4
P41	0.67	0.54	80.6
P42	1.9	2.3	121.05
P43	1.69	1.36	80.47
P44	4.48	5.3	118.3
P45	4.67	4.9	104.93
P46	5	5.17	103.4
P47	1.51	1.8	119.21
P48	3.75	4.3	114.67
P49	2.98	3.12	104.7
P50	3.37	3.42	101.48
P51	1.61	1.31	81.37
P52	4.03	3.72	92.31
P53	7.4	7.87	106.35
P54	2.31	2.8	121.21
P55	1.72	1.73	100.58
P56	3.31	2.94	88.82
P57	3.72	2.97	79.84
P58	2.6	3	115.38
P59	2.65	2.63	99.25
P60	2.93	2.42	82.59
P61	1.14	0.99	86.84
P62	1.6	1.62	101.25
P63	0.81	0.76	93.83
P64	4.53	3.92	86.53
P65	1.23	1.17	95.12
P66	4.74	3.95	83.33

P67	1.46	1.22	83.56
P68	1.48	1.18	79.73
P69A	1.15	1.13	98.26
P69B	3.73	3.91	104.83
P70	1.68	1.91	113.69
P71	1.12	1.15	102.68
P72	1.27	1.14	89.76
P73	1.33	1.38	103.76
P74	2.03	2.17	106.9
P75	2.82	2.69	95.39
P76	1.2	1.15	95.83
P77	2.39	2.31	96.65
P78	0.7	0.61	87.14
P80	0.88	0.89	101.14
P81	1.86	2.03	109.14
P82	1.07	1.1	102.8
P83	3.35	2.84	84.78
P84	2.78	3.2	115.11
P85	0.91	0.86	94.51
P86	3.7	3.5	94.59
P87	1.18	1.21	102.54
P88	0.7	0.61	87.14
P89	1.24	1.38	111.29
P90	1.03	1.19	115.53
P91	1.57	1.32	84.08
P92	2.58	2.93	113.57
P93	2.22	2.34	105.41

Table S2: Blood samples recovery with developed LFA device.

Patient Number	Cystatin-C, Atellica-NEPH-630 (µg/mL)	Cystatin-C, LFA (µg/mL)	Recovery (%)
P59	2.65	2.7	101.89
P61	1.14	1.39	121.93
P62	1.6	1.72	107.5
P63	0.81	0.83	102.47
P64	4.53	5.14	113.47
P65	1.23	1.3	105.69
P66	4.74	5.51	116.24
P67	1.46	1.52	104.11
P68	1.48	1.76	118.92
P69A	1.15	1.11	96.52
P69B	3.73	4.44	119.03
P70	1.68	1.47	87.5
P72	1.27	1.16	91.34
P73	1.33	1.56	117.29
P75	2.82	2.7	95.74
P76	1.2	0.98	81.67
P77	2.39	2.68	112.13
P79	1.38	1.4	101.45
P83	3.35	4.11	122.69
P92	2.58	2.5	96.9
P93	2.22	2.22	100
